# A case of strongly suspected Lynch syndrome with colorectal neuroendocrine carcinoma

**DOI:** 10.1186/s40792-022-01471-0

**Published:** 2022-06-17

**Authors:** Naoya Kobayashi, Hiroshi Yoshida, Shinya Kawaguchi, Satoru Shiraso, Noriko Nemoto, Nanako Fujikawa, Yoichi Haji, Emiko Kono, Shoji Kokubo, Kazuhiko Tsukuda, Shigeyuki Asano, Fumiaki Shinya

**Affiliations:** 1 Department of Surgery, Iwaki City Medical Center, 16 Kusehara, Uchigomimayamachi, Iwaki-shi, Fukushima 973-8555 Japan; 2 Department of Pathology, Iwaki City Medical Center, Iwaki-shi, Japan

**Keywords:** Neuroendocrine cell carcinoma, Colorectal cancer, Lynch syndrome

## Abstract

**Background:**

Neuroendocrine cell carcinomas (NEC) of the colon and rectum are uncommon, representing ~ 0.1% of all colorectal carcinomas. They are associated with a much worse prognosis compared to adenocarcinoma of the colon and rectum, as death occurs in approximately half of all patients within 1 year. Lynch syndrome (LS) is the most common cause of inherited colorectal cancer, accounting for 2–4% of newly diagnosed colorectal cancer cases. This case is extremely rare which was strongly suspected LS as the background, and NEC as the histological type of colorectal cancer.

**Case presentation:**

The patient was a 44-year-old man presenting with vomiting as the main complaint. He had undergone ileocecal resection for cecal cancer at age 29. The diagnosis was obstructive descending colorectal cancer, and colonoscopy revealed tumors in the rectum and sigmoid colon in addition. Due to multiple occurrences of colorectal cancer and its prevalence in the patient’s family, LS was suspected. The operation which was a subtotal proctocolectomy was performed. Pathological analysis revealed complete curative resection and the descending colon cancer of the obstructed portion was at the most advanced pathological Stage IIIC in UICC TNM classification, and the tissue type was a NEC. The Ki-67 index was 70%. The results of the microsatellite instability (MSI) test showed high-frequency MSI. The BRAF V600E variant was negative. The immunoexpression of MLH1 was positive, MSH2 was negative, PMS2 was positive, and MSH6 was negative.

**Conclusions:**

Extended surgery is recommended for incipient colorectal cancer in LS cases in order to reliably reduce the risk of developing metachronous colorectal cancer. The survival outcome of surgery alone on digestive tract NECs, even locoregional lesions that are completely resection, is extremely poor. It is currently unclear if digestive tract NECs develop more readily in patients with LS. The accumulation of additional cases is necessary.

## Background

Neuroendocrine cell carcinomas (NEC) of the colon and rectum are uncommon, representing ~ 0.1% of all colorectal carcinomas [1, 2]. They are associated with a much worse prognosis compared to adenocarcinoma of the colon and rectum, as death occurs in approximately half of all patients within 1 year [3–6]. In the 2019 World Health Organization (WHO) classification, neuroendocrine tumors are staged on the basis of differentiation, grade, mitotic rate, and Ki-67 index [7].

Lynch syndrome (LS) is the most common cause of inherited colorectal cancer, accounting for 2–4% of newly diagnosed colorectal cancer cases [8]. This syndrome is characterized not only by colorectal cancer, but also by common development of extracolonic malignancies, including cancers in the endometrium, ovaries, gastrointestinal tract, and urinary tract [9–12]. This hereditary cancer syndrome is transmitted through an autosomal dominant pattern caused by germline mutations in the mismatch repair (MMR) genes MLH1, MSH2, MSH6, and PMS2, or in the epithelial-cell adhesion molecule gene [13, 14].

We report our experience with a patient who was strongly suspected to have LS with NEC.

## Case presentation

**Fig. 1:**
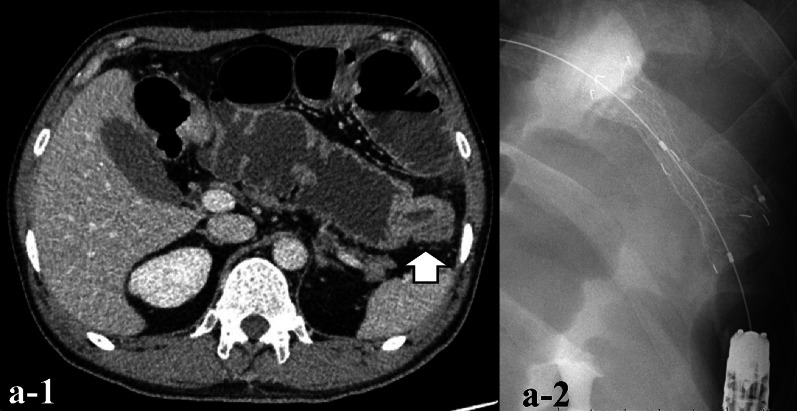
Abdominal CT and a colon stent. **a-1** Contrast-enhanced peripheral wall enhancement (white arrow) was observed in the descending colon and splenic flexure. Dilation of the gastrointestinal tract was also observed. **a-2** A colon stent was placed at the same site

**Fig. 2:**
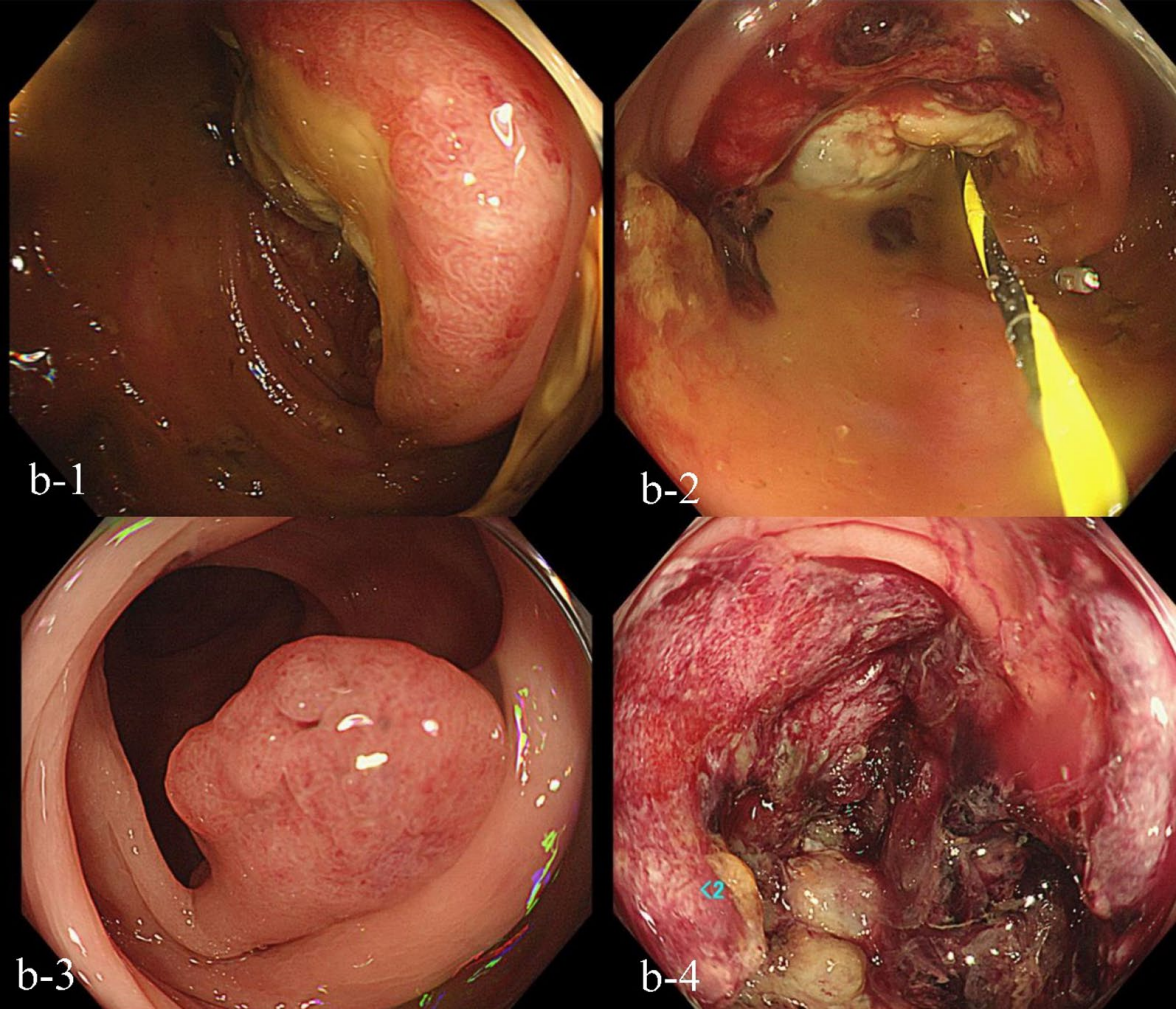
Colonoscopy findings. **b-1** A circumferential type 2 tumor was observed in the descending colon. Categorized into Group 5 after biopsy. **b-2** A colon stent was placed at the same site. **b-3** A polyp was observed in the sigmoid colon. **b-4** A type II tumor with a 75% circumference was observed in the rectum. Categorized into Group 5 after biopsy

**Fig. 3:**
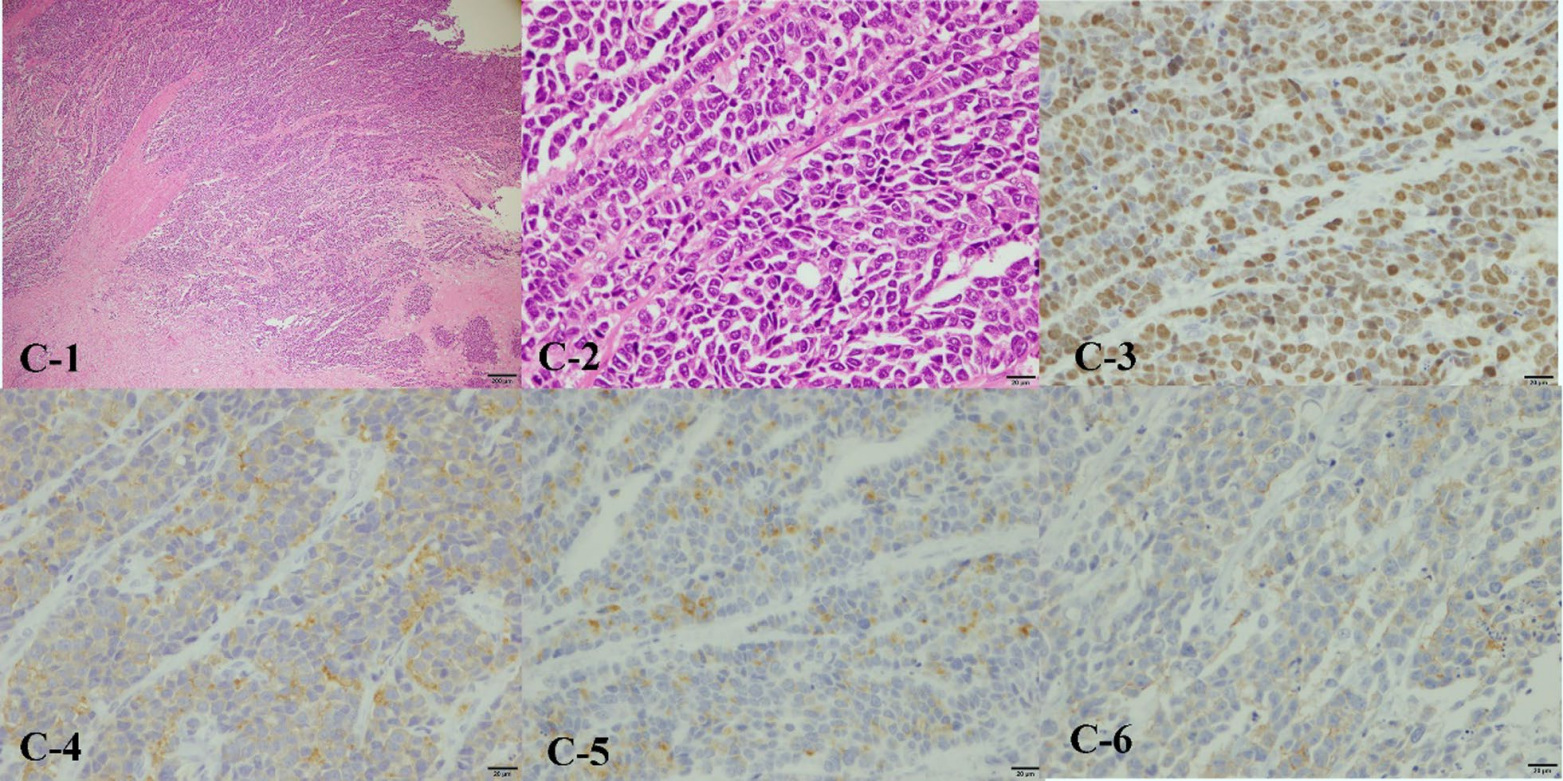
Pathological analysis. **c-1-2** Greatly increased anisonucleosis and a high mitotic index were observed by H&E staining. A partial cord-like sequence was observed. **c-3** Ki-67 was more than 70% positive. **c-4** Synaptophysin was positive by immunostaining. **c-5** Chromogranin A was positive by immunostaining. **c-6** CD 56 was positive by immunostaining

The patient was a 44-year-old man presenting with vomiting as the main complaint. He had undergone ileocecal resection for cecal cancer at age 29. Abdominal CT showed thickening of the wall of the descending colon and dilation of the digestive tract. The diagnosis was obstructive descending colon cancer, and a colonic stent was inserted (Fig. 1). A colonoscopy revealed tumors in the rectum and sigmoid colon in addition (Fig. 2). Due to multiple occurrences of colorectal cancer and its prevalence in the patient’s family (his grandfather on his father’s side and mother died due to colon cancer, and his father underwent colon cancer at 49 years of age, and died due to duodenal cancer), LS was suspected. The operation which was a subtotal proctocolectomy with ileo-anal canal anastomosis and a temporary loop ileostomy were performed. Ascending colon cancer was also observed during surgery. Pathological analysis was complete curative resection and the descending colon cancer of the obstructed portion was at the most advanced pathological Stage IIIC in UICC TNM classification, and the tissue type was NEC. The tissue type of other lesions of rectal, sigmoid and ascending colon was adenocarcinoma, either. Anisonucleosis and mitosis of the NEC were observed by H&E staining. The Ki-67 index of the NEC was 70%, which suggests proliferative activity. By immunostaining, chromogranin A, synaptophysin, and CD56, which are all characteristic markers of NEC or neuroendocrine tumor (NET), were positive. CK7 was negative and CK20 was positive, indicating the NEC originating from colon (Fig. 3). SSTR2 expression was negative. The results of the microsatellite instability (MSI) test showed high-frequency MSI. The BRAF V600E variant was negative. The immunoexpression of MLH1 was positive, MSH2 was negative, PMS2 was positive, and MSH6 was negative. And LS was strongly suspected as was expected. Adjuvant chemotherapy was administered by cisplatin and etoposide combination. The patient developed hypomagnesemia, and chemotherapy was halted after three courses. At 6 months postoperatively, there was no relapse and made for surgical closure of the ileostomy.

## Discussion

There are options in the extent of colon resection when colorectal cancer caused due to LS. The extent of resection could be the same as would be chosen for sporadic colorectal cancer, or extended surgery (total colectomy, subtotal colectomy) could be performed [15]. Extended surgery is recommended for incipient colorectal cancer in LS cases in order to reliably reduce the risk of developing metachronous colorectal cancer [15]. In this case, the operation was performed by a subtotal proctocolectomy. In the meta-analysis, metachronous colorectal cancer developed in 22.4–22.8% of cases with partial resection of the colon and in 4.7–6.8% of cases with extended surgery, which shows that partial resection of the colon significantly increases the risk of developing metachronous colorectal cancer in LS [16].

This case fulfills the Amsterdam criteria II, as described below. At least three relatives (the patient’s father, mother, and grandfather) had with a hereditary nonpolyposis colorectal cancer (HNPCC)-associated cancer (colorectal cancer, cancer of endometrium, small bowel, ureter, or pelvis). The patient is a first-degree relative of the two, and two successive generations are affected. The patient diagnosed before age 50 years. Familial adenomatous polyposis was excluded in the colorectal cancer case. Tumor was verified by pathological examination [17]. And, by the revised Bethesda guidelines, this case should be tested for MSI, because the patient was in the following situation, as described below. In this case, colorectal cancer diagnosed in the patient who was less than 50 years of age. It is presence of synchronous, metachronous colorectal tumors. Colorectal cancer was diagnosed in one or more first-degree relatives (his father and mother) with an HNPCC-related tumor, and the patient and his father were diagnosed under age 50 years. Colorectal cancer was diagnosed in two or more first- or second-degree relatives (his father, mother, and grandfather) with HNPCC-related tumors, regardless of age [18].

**Fig. 4:**
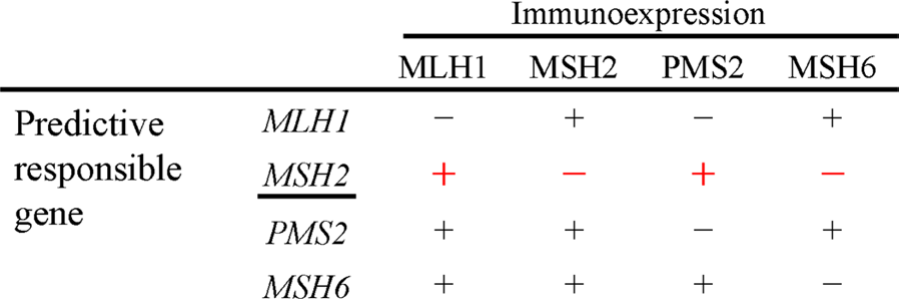
The pattern of immunohistochemical staining for MMR protein. The red line was the pattern of this case. And the pattern suggested that predictive responsible gene of this case was MSH2

It is important to rule out the possibility that the tumor of this patient might be MSI-high sporadic colorectal cancer for the diagnosis of LS. BRAF V600E variant gene rarely found in the case of LS even if MSI-high, and the testing of BRAF V600E variant gene has possibility to help the diagnosis of LS [19]. In this case, the BRAF V600E variant of the patient was negative, and it is not contradiction as LS. In this case, the pattern of immunohistochemical staining for MMR protein of MLH1, MSH2, PMS2, and MSH6 suggested that predictive responsible gene was MSH2 (Fig. 4) [20]. Regarding the diagnosis of LS, it is essential to identify pathological germline MMR genes, and to prove that this patient has the germline MMR genes mutation [13, 14]. In this case, we recommended the patient to receive genetic counseling and the testing of the germline MMR genes at the specialized organization, and the patient went to consult genetic professional doctor and got genetic counseling. The patient was recommended the testing of the germline MMR genes. However, the testing is out of the insurance coverage, then, the patient refused to take the testing.

The tissue type of the descending colon cancer was NEC in this case, and the Ki-67 index was > 70%. Mitosis was observed in both the NET and NEC, but it was seen to a much greater extent in NEC. The Ki-67 index exceeded 20% in NET and NEC, but NEC tended to show abnormally high values in excess of 50%. With regard to SSTR2 expression, clear positive staining was observed in a non-localized manner in NET; however, in NEC, the findings were either weakly positive or negative [21]. The overall outcome of surgery alone on digestive tract NECs, even locoregional lesions that are completely excised, is extremely poor; the reported median overall survival is 14.7 months [22]. The median overall survival of the group under which the operation with multidisciplinary treatment such as perioperative chemotherapy and radiation therapy was slightly better than that of the operation alone group (20.4 months vs. 15.4 months, *P* = 0.08) [22]. With regard to postoperative adjuvant chemotherapy of digestive tract NECs, that evidence still have been not strong today. However, according to the guideline of NCCN (National comprehensive cancer network), ENETS (European neuroendocrine tumor society) and NANETS (North American neuroendocrine tumor society), cisplatin + etoposide, or cisplatin + irinotecan which are typical therapeutic regimens of small cell lung cancer are recommended for postoperative adjuvant chemotherapy of the digestive tract NECs with four to six courses [23–25].

## Conclusions

This case is extremely rare which was strongly suspected LS as the background, and NEC as the histological type of colorectal cancer. It is currently unclear if digestive tract NECs develop more readily in patients with colorectal cancer with LS. The accumulation of additional cases is necessary.

## Data Availability

Not applicable.

## Abbreviations

NEC: Neuroendocrine cell carcinomas
NET: Neuroendocrine tumor
LS: Lynch syndrome
MMR: Mismatch repair
MSI: Microsatellite instability
HNPCC: Hereditary nonpolyposis colorectal cancer
